# Performance of Mycobacterium Growth Indicator Tube BACTEC 960 with Lowenstein–Jensen method for diagnosis of *Mycobacterium tuberculosis* at Ethiopian National Tuberculosis Reference Laboratory, Addis Ababa, Ethiopia

**DOI:** 10.1186/s13104-017-2497-9

**Published:** 2017-05-10

**Authors:** Getu Diriba, Abebaw Kebede, Zelalem Yaregal, Muluwork Getahun, Mengistu Tadesse, Abyot Meaza, Zekarias Dagne, Shewki Moga, Jibril Dilebo, Kebebe Gudena, Mulu Hassen, Kassu Desta

**Affiliations:** 1 Ethiopian Public Health Institute, Addis Ababa, Ethiopia; 2 School of Laboratory Science, College of Allied Science, Addis Ababa University, Addis Ababa, Ethiopia; 3 Department of Laboratory Science, College of Health Sciences, Addis Ababa University, Addis Ababa, Ethiopia

**Keywords:** Tuberculosis, Lowenstein–Jensen, *Mycobacteria* Growth Indicator Tube

## Abstract

**Background:**

Bacteriological confirmed active case detection remains the corner stone for diagnosing tuberculosis. Non-radiometric liquid culture system *Mycobacterium* Growth Indicator Tube with automated interface had been recommended by expert groups in addition to conventional solid culture media such as Lowenstein–Jensen. However in high burden resource limited countries advanced non-radiometric based tuberculosis diagnostic methods such as MGIT 960 is limited. Therefore we have evaluated the performance of MGIT 960 system compared to LJ for recovery of *Mycobacterium* complex (MTBC) from clinical specimens.

**Methods:**

A cross sectional study was conducted from a total of 908 samples between January 1st, 2013 to December 31st, 2014. Clinical specimens were processed following standard procedures and the final suspension was inoculated to MGIT tubes and LJ slant. Identification and confirmation of MTBC was done by ZN staining and SD Bioline test. Data was analyzed by SPSS version 20. The sensitivity, specificity, recovery rate and the average turnaround time to recover the organism was computed.

**Results:**

From a total of 908 clinical specimens processed using both LJ and BACTEC MGIT liquid culture methods the recovery rate for LJ and MGIT, for smear positive samples was 66.7% (74/111) and 87.4% (97/ 111) respectively while for smear negative samples was 13.4% (108/797) and 17.4% (139/797) for LJ and MGIT methods respectively. The overall recovery rate for MGIT is significantly higher than LJ methods [26% (236/908; vs. 20%, 182/908, *P* = *0.002*)]. The average turnaround time for smear positive samples was 16 and 31 days for MGIT and LJ respectively. Turnaround time for smear negative samples was 20 and 36 days for MGIT and LJ respectively. The overall agreement between MGIT and LJ was fairly good with Kappa value of 0.59 (P < 0.001). In the present study the contamination rate for MGIT is higher than the LJ methods, 15 and 9.3% respectively.

**Conclusions:**

The BACTEC MGIT liquid culture system has better MTBC recovery rate with shorter turnaround time for both smear positive and negative clinical specimens compared to Conventional LJ method. However, efforts should be made in order to reduce the high contamination rate in BACTEC MGIT system and to lesser extent to LJ methods.

## Background

Symptomatic active form of TB is the most crucial factor for transmission of infection. Even though competent immune system will limit the multiplication, some bacilli may remain dormant latently. Relatively, small proportion of exposed people develops TB disease at any course of their life; the probability of developing TB is much higher among people infected with HIV [1]. Burden of tuberculosis in developing countries remain high and affecting the most productive young ages as a result of an enormous economic lose in the populations of most low- income countries of Asia and Africa which were evident from many reports [2].

Bacteriological confirmed active case detection remains the corner stone for diagnosis of Tuberculosis, However, among the most utilized diagnostics methods; TB smear microscopy is the most popular among all available methods in developing countries. In addition, most commonly used diagnostic technique such as LJ based myco-bacteriological culture and recently introduced TB molecular diagnostic technique with efficient turnaround time and specificity is also used for the diagnosis of active forms of TB in clinical settings [3].

The most popular LJ based myco-bacteriological culture TB diagnostic methods, Lowenstein–Jensen (LJ), is time consuming, taking up to 6–8 weeks to recover *Mycobacteria* or discriminate negative samples [4, 5].

Non-radiometric liquid culture methods called *Mycobacterium* Growth Indicator Tube (MGIT; Becton Dickinson) system have been introduced for better recovery of *Mycobacterium* [6]. This MGIT 960 system is a fully automated, non-radiometric, and used to incubate and monitor 960 samples at a time with automated result-reporting system [7]. The methods designed to shorten turnaround time with good recovery rate than conventional solid TB culture system [8]. The MGIT 960 system has been in place of the national tuberculosis laboratories of Ethiopia that is sued along with the conventional LJ methods. However, there overall performance of MGIT is not known for large samples. Hence, we aimed to evaluate the performance of BACTEC MGIT 960 system in comparison with Lowenstein–Jensen culture methods in Ethiopian context hoping that the finding could supplement TB diagnostic methods in high TB burden resource poor countries.

## Methods and materials

A cross sectional study was conducted from total 908 TB suspected patients who were referred for sputum culture testing at Ethiopian Public Health Institute (EPHI), National TB Reference laboratory from January 1st, 2013–December 31st, 2014.

### Laboratory methodology

Specimens were processed by the sodium hydroxide and N-acetyl-l-cysteine (NaOH/NALC) method and concentrated by centrifugation (3000 RPM for 15 min). The supernatant was discarded, and the sediment was re suspended with sterile phosphate buffer to a final volume of 2 ml. This suspension was then used for making smears for acid-fast staining and for inoculation in to BACTEC MGIT 960 culture tube (0.5 ml) and LJ slant (2–3 drops). In most cases, the specimens were processed on the day of collection. Those that could not be processed on the day of collection (i.e., received during the weekend) were stored at 2–8 °C for 2 days. Samples collected and received more than 3 days were rejected based on the laboratory rejection criteria and documented on sample rejection log book. Smears were examined for acid-fast bacilli (AFB). All AFB smears were stained by the Ziehl–Neelsen staining method and examined with a bright filed microscope following the standard guidelines of our laboratory.

### Culture of *Mycobacteria*

#### BACTEC MGIT 960 system

The BACTEC MGIT 960 culture tube contains 7 ml Middle brook 7H10 broth base, in which an enrichment supplement containing oleic acid, albumin, dextrose, and catalase is added. In addition, an antibiotic mixture of polymyxin B, amphotericin B, nalidixic acid, trimethoprim, and azlocillin is added to the culture tube to inhibit growth of other microbes.

The culture tube contains a fluorescent sensor that detects the concentration of oxygen in the culture medium. The level of fluorescence corresponds to the amount of oxygen consumed by the organisms in the inoculated specimens. This, in turn, is proportional to the number of bacteria present. When a certain level of fluorescence is reached, the instrument indicated that the tube is positive. After inoculation of each tube with 0.5 ml of the processed specimen, the tubes were incubated at 37 °C in the BACTEC MGIT 960 instrument and monitored automatically every 60 min for increased fluorescence. The culture tubes were maintained until it became positive or for 42 days of maximum for negative samples. Samples found to be positive were removed from the instrument then sub cultured on Brain Heart Infusion agar plate, incubated for 48 h at 37 °C to asses possible contaminants. All the positive tubes were further confirmed by ZN staining methods and further confirmed by MPT64 protein Specific detection immune chromatographic test (SD Bioline Kit, Standard Diagnostics, Inc., Korea). The kit can discriminate MTBC and non *Mycobacterium tuberculosis* complex.

#### Solid media

All specimens which were processed were also inoculated onto conventional solid media. LJ slant. The LJ slant medium was considered positive upon appearance of colonies on the surface, and the time to detection was based on the earliest date of detection of colonies on the solid media, ultimately confirmed by positive AFB smears and MPT64 protein specific detection immuno chromatographic test (SD Bioline Kit, Standard Diagnostics, Inc., Korea). Solid media were incubated at 37 °C for 8 weeks, and were inspected twice weekly or until Mycobacterium colonies were seen.

## Results

A total of 908 patients samples were included for this evaluation purpose. In this study, 423 (46.6%) were new patients refereed to our laboratory and 485 (53.4%) patients were follow up patients that are refereed for Rifampicin and Isoniazid Resistance testing (Table 1).

**Table 1:** Patient diagnostic categories with respective to age group at EPHI/NTRL since 2015

Age group	Category of patient		Total no. (%)
	New patient	MDR-TB follow up patient	
4–16	28 (3.1%)	21 (2.3%)	49 (5.4%)
17–29	127 (14%)	262 (28.9%)	389 (42.8%)
30–42	148 (16.3%)	160 (17.6%)	308 (33.9%)
43–55	62 (6.8%)	30 (3.3%)	92 (10%)
56–68	47 (5.2%)	9 (1%)	56 (6.2%)
69–80	11 (1.2%)	3 (0.3%)	14 (1.5%)
Total	423 (46.6%)	485 (53.4%)	908 (100%)

The overall recovery rate of MTBC by BACTTEC MGIT 960 method was 14.4% (131/908) and 11.5% (105/908) for new TB patients and for MDR-TB follow up patients respectively. On the other hand, the conventional LJ methods recovered relatively lower no. MTBC cases, 11.3% (103/908) and 8.7% (79/908) respectively for new and MDR TB follow up patients.

Overall, 26% of MTBC was detected by BACTEC MGIT methods while LJ methods detected 20% (182/908) of MTBC cases. A total of 534 (58.8%) specimens were negative by either LJ or MGIT methods and 138 (15%) specimens were contaminated (Table 2). Relatively good agreement has been observed between BACTEC MGIT 960 and LJ methods with Kappa value of 0.59.

**Table 2:** Comparison of MGIT and LJ methods for MTBC recovery and contamination rate at EPHI/NTRL since 2015

	LJ result (%)			Total
	Positive	Negative	Contaminated	
MIGIT result				
Positive no. (%)	171 (18.8%)	58 (6.4%)	7 (0.8%)	236 (25.9%)
Negative no. (%)	7 (0.8%)	497 (54.7%)	30 (3.3%)	534 (58.8%)
Contaminated no. (%)	4 (0.44%)	87 (9.6%)	47 (5.2%)	138 (15%)
Total	182 (20%)	642 (70.7%)	84 (9.3%)	908 (100%)

In this study, 139 (17.4%) and 108 (13.6%) of smear negative cases were found to be positive for MTBC using BACTEC MGIT 960 and LJ culture methods respectively. The recovery rate of BACTEC MGIT was found significantly higher than LJ method (P < 0.007). The overall agreement for MTBC detection between BACTEC MGIT 960 method and AFB smears method were 87.4%. Whereas the agreement between LJ culture methods and AFB smears were low, only 66.7% (Table 3).

**Table 3:** Performance and contamination of BACTEC MGIT 960 and LJ methods along with smear status at EPHI/NTRL since 2015

	MGIT				LJ			
	Positive	Negative	Contaminated	Total	Positive	Negative	Contaminated	Total
Smear positive	97 (87.4%)	13 (11.7%)	1 (0.9%)	111 (100%)	74 (66.7%)	33 (29.7%)	4 (3.6%)	111 (100%)
Smear negative	139 (17.4%)	521 (65.4%)	137 (17.2%)	797 (100%)	108 (13.6%)	609 (76.4%)	80 (10%)	797 (100%)

BACTEC MGIT 960 system has higher contamination rate than LJ methods, 15% and 9.2 respectively. The contamination rate of MGIT 960 system and LJ method for new patients were 6.7 and 4% respectively (Table 4).

**Table 4:** Patient categories and performance of BACTEC MGIT 960 and LJ methods for diagnosis of *Mycobacterium tuberculosis* infection at EPHI/NTRL since 2015

	MGIT				LJ			
	Positive	Negative	contaminated	Total	Positive	Negative	Contaminated	Total
New patient	131 (14.4%)	230 (25.3%)	62 (6.7%)	423 (46.5%)	103 (11.3%)	283 (31.2%)	37 (4%)	423 (46.9)
MDR-TB follow up patient	105 (11.6%)	304 (33.5%)	76 (8.3%)	485 (53.5%)	79 (8.7%)	359 (39.8%)	47 (5.2%)	485 (53.4%)

The average time to detect (TTD) significant growth of *Mycobcateria* from smear-positive specimens using BACTEC MGIT 960 was 16 days and 20 days for smear-negative patients. Whereas TTD using LJ method was 31 days for smear positive cases and 36 days for smear negative specimens. There is significant difference between culture methods and TTD, MGIT picked MTBC cases faster than LJ methods both for smear positive and smear negative cases (P < 0.001).

## Discussion

Non radiometric *Mycobacterium* Growth Indicator Tube Liquid culture is one of TB culture method which is endorsed and recommended methods by World Health Organization. The method is found to be rapid and has 10% improved sensitivity for recovery of MTBC over the conventional egg based LJ method. However, its utilization is limited to only in few high burden resources limited countries including Ethiopia. Hence in order to enhance the applicability of this method under resource limited settings, the present study was evaluated and significant difference has been observed between the BACTEC MGIT 960 and LJ methods interims of recovery rate and time of detection.

Improved recovery rate of *Mycobacterium* species were observed compared to the conventional LJ method with significant shorter turnaround time (TAT) for both smear positive and Negative sputum specimens (87.4 vs. 66.7% for MGIT and LJ methods respectively). This finding is comparable with similar study from Nigeria for smear positive specimen using BACTEC MGIT 960 system with recovery rate of 87% but improved recovery rate were reported using LJ method with the rate of 78% than our finding [9]. Comparable findings were reported in USA with the rate of 82% for BACTEC MGIT and 76% with LJ method [10]. South African study reported almost similar finding with recovery rate of 83% using BACTEC MGIT for smear positive specimens [11].

Higher recovery rates were reported in Taiwan from smear positive specimen with 100% using BACTEC MGIT 960 system and 92.9% with LJ method [6]. Similarly, higher recovery rates were reported from Germany, Turkey, Yugoslavia, Hungary and Spain from smear positive specimen with rates of 94.7% and 94.7, 88 and 83%, 93.2 and 67.3%, 96.4 and 81.8%, 95.5 and 79.5% for BACTEC MGIT and LJ methods respectively [14–17]. Unlike the present study, a much higher recovery rate was reported from Iraq using similar platform with the rate of 100% for MGIT 960 and 72.6% for LJ method [19]. A study from Portland, Oregon also reported a recovery rate of 100 and 90.3% for BACTEC MGIT 960 AND LJ method respectively [13]. Another studies from Pakistan, also reported higher recovery rate of 97.6 and 83.7% for BACTEC MGIT and LJ respectively [12]. On the other hand, lower recovery rate was reported from USA and Hungary with the rate of 85.5 and 59.7%, 81 and 64.3%, for BACTEC MGIT and LJ methods respectively [8, 21]. The most likely difference between our findings with the aforementioned report could be difference in sample size, burden of tuberculosis and the sample size.

In this study the recovery rate for smear negative samples using BACTEC MGIT method were 17. 4% (139/797) compared 13.6% (108/797) for LJ method. This observation is in consistent with the findings of previous study in Nigeria that reported a recovery rate of 17 and 11% for MGIT and LJ method respectively [9].

Our finding is lower than report from smear negative Iraqi patients, with recovery rate of 24.2 and 14.7% for BACTEC MGIT and LJ method respectively [19]. Australian workers reported a recovery rate of 21.8 and 19.4% [20] for MGIT and LJ method respectively.

Relatively lower recovery rate from smear negative patients was reported from USA and Hungary with the rate of 14.3 and 10%, 16.6 and 6.6% for BACTEC MGIT and LJ respectively [8, 21]. The recovery rate difference from smear positive and negative specimen might be associated with number of viable AFB inoculated into MGIT tube, the type of species of *Mycobacteria*, such as *M. tuberculosis and Mycobacterium bovis* that grow more slowly than NTM, such as *Mycobacterium avium* complex. It also depends on certain types of specimen quality. Another important factor that can lead to variable growth rate was the treatment status of patients. Particularly Specimens from chronically treated patients with drug resistant TB take a longer time to grow. This is true in our case, were more than 50% of the specimens were processed for MDR TB follow up patients.

Irrespective of the pre analytical variable mentioned so far, procedure and methodology used, potentially affects growth rate. Especially high pH or very low pH may cause injury or death to *Mycobacteria* during processing of the specimen. As a result it takes longer TAT for revival and growth of viable *Mycobacteria*. Some researcher reported that as many as 60–70% of the *Mycobacteria* are killed during sputum processing and centrifugation [18, 28, 32].

Another key feature of a culture method is contamination rate, in our study we found high contamination rate compared to pre-established standard of 5–8% for liquid culture and 3–5% for solid LJ culture methods [28], according to our finding, both BACTEC MGIT 960 and LJ methods had high contamination rates (15 and 9.3% respectively) . Similar findings has been reported from U.S.A, Taiwan, Spain and Turkey under similar evaluation conditions with contamination rates of 17, 15.1, 13.6, 12, 9% for the BACTEC MGIT method respectively [6, 22–25]. Unlike our study findings acceptable contamination rate were reported from Nigeria, Iraq and Germany with the rate of 7, 4.8 and 8.1% respectively for BACTEC MGIT method [11, 19, 26]. The discrepancy could be explained due to problems in sample collection, transportation, patient instructions, rejection criteria of the laboratory and difference in sample storage conditions [28].

Average time to detect minimum positive growth unit (70 Colony Forming Units of *M. tuberculosis*) using BACTEC MGIT 960 from smear-positive specimens were 16 days compared to 31 days for manual LJ methods. Comparable TTD for smear positive specimen was reported from Yugoslavia and India with average TTD time for BACTEC MGIT and LJ method of 13.7 days and LJ 22.1 days; 13.1 and 23.9 days respectively [13, 16]. Shorter recovery days were reported by Luqman Satti et al. from Pakistan with TTD of 11.2 days for BACTEC MGIT system [12]. Similarly, shorter TTD was also reported from Taiwan and Turkey with average TTD days of 9.1 and 17.6 for BACTEC MGIT and 8.3 and 20.1 days for LJ methods [6, 25].

In our study a mean TTD, 20 days was recorded using BACTEC MGIT and 36 days using LJ method for smear negative patients Comparable findings were also reported by Piersimoni et al. in Malaysian and Italian researchers with TTD for MGIT/ LJ of 20/33.1, 13.2/35.3 and 13.3/25.6 days respectively [27, 29, 30]. The variation between studies could be due to the nature of patient categories and other factors such as low bacillary load from treatment groups [28].

In this study 13 (1.4%) and 33 (3.6%) specimens were smear-positive but culture-negative based on BACTEC MGIT and LJ methods respectively. Higher rate of 3.2 and 5.4% were reported for BACTEC MGIT and LJ method respectively [8]; some study also reported 6% each for BACTEC MGIT and LJ method [31]. Very high rates were reported for smear positive cases with the rate of 6.5% by BACTEC MGIT and 9.2% by LJ methods [16]. This discrepancy might be associated with factor such as harsh method used to decontaminate the specimen or prolonged exposure of decontaminant, NaOH during processing [18]. It could be also associated with media quality and composition [28]. Additional factors like good laboratory practice to follow and inspect the quality control methods for every batch of culture media and specimen may also contribute for differences [30].

## Conclusions

We have observed that the overall performance of BACTEC MGIT liquid culture system is found to be better than the conventional LJ methods for rapid recovery of *M. tuberculosis* complex with shorter turnaround time for both smear positive and negative clinical specimens. However in efforts should be made in order to minimize the contamination rate of both BACTEC MGIT and LJ methods. Contentious improvement plan is required for provision of quality TB laboratory services which are crucial for control and eradication of tuberculosis at large.

## Abbreviations

AFB: acid-fast bacilli
DST: drug sensitivity testing
EPHI: Ethiopian Public Health Institute
LJ: Lowenstein–Jensen
MDR-TB: multi-drug-resistant tuberculosis
MGIT: *Mycobacterium* Growth Indicator Tube
MTBC: *Mycobacterium tuberculosis* complex
NTM: *non*-*tubercles Mycobacterium*
NTRL: National Tuberculosis Reference Laboratory
PTB: pulmonary tuberculosis
RPM: revolution per minutes
TAT: turnaround time
TB: tuberculosis
TTD: time to detection
ZN: Ziehl–Neelsen
